# Barotrauma during Noninvasive Respiratory Support in COVID-19 Pneumonia Outside ICU: The Ancillary COVIMIX-2 Study

**DOI:** 10.3390/jcm12113675

**Published:** 2023-05-25

**Authors:** Luigi Vetrugno, Cristian Deana, Nadia Castaldo, Alberto Fantin, Alessandro Belletti, Emanuela Sozio, Maria De Martino, Miriam Isola, Diego Palumbo, Federico Longhini, Gianmaria Cammarota, Savino Spadaro, Salvatore Maurizio Maggiore, Flavio Bassi, Carlo Tascini, Vincenzo Patruno, COVIMIX-STUDY Group

**Affiliations:** 1 Department of Medical, Oral and Biotechnological Sciences, University of Chieti-Pescara, 66100 Chieti, Italy; luigi.vetrugno@unich.it; 2 Department of Anesthesiology, Critical Care Medicine and Emergency, SS. Annunziata Hospital, 66100 Chieti, Italy; salvatore.maggiore@unich.it; 3 Department of Anesthesia and Intensive Care, Health Integrated Agency of Friuli Venezia Giulia, Piazzale Santa Maria della Misericordia 15, 33100 Udine, Italy; flavio.bassi@asufc.sanita.fvg.it; 4 Pulmonology Unit, Department of Cardio-Thoracic Surgery, Health Integrated Agency of Friuli Venezia Giulia, 33100 Udine, Italy; nadiacastaldo.nc@gmail.com (N.C.); af@albertofantin.com (A.F.); vincenzo.patruno@asufc.sanita.fvg.it (V.P.); 5 Department of Anesthesia and Intensive Care, IRCCS San Raffaele Scientific Institute, 20132 Milan, Italy; belletti.ale@gmail.com; 6 Infectious Disease Unit, Health Integrated Agency of Friuli Venezia Giulia, 33100 Udine, Italy; emanuela.sozio@gmail.com (E.S.); carlo.tascini@uniud.it (C.T.); 7 Department of Medical Area, University of Udine, 33100 Udine, Italy; maria.demartino@uniud.it (M.D.M.); miriam.isola@uniud.it (M.I.); 8 Department of Radiology, IRCCS San Raffaele Scientific Institute, 20132 Milan, Italy; palumbo.diego@hsr.it; 9 Anesthesia and Intensive Care Unit, Department of Medical and Surgical Sciences, University Hospital Mater, Domini, Magna Graecia University, 88100 Catanzaro, Italy; longhini.federico@gmail.com; 10 Anesthesiology and Intensive Care, Department of Translational medicine, Faculty of Medicine and Surgery, University of Ferrara, 44121 Ferrara, Italy; gianmaria.cammarota@unipg.it; 11 Department of Medicine and Surgery, University of Perugia, 06123 Perugia, Italy; savinospadaro@gmail.com; 12 Department of Innovative Technologies in Medicine and Dentistry, Gabriele d’Annunzio University of Chieti Pescara, 66100 Chieti, Italy

**Keywords:** barotrauma, noninvasive ventilation, COVID-19, pneumothorax, high flow nasal oxygen, acute respiratory failure

## Abstract

Background: Noninvasive respiratory support (NIRS) has been extensively used during the COVID-19 surge for patients with acute respiratory failure. However, little data are available about barotrauma during NIRS in patients treated outside the intensive care unit (ICU) setting. Methods: COVIMIX-2 was an ancillary analysis of the previous COVIMIX study, a large multicenter observational work investigating the frequencies of barotrauma (i.e., pneumothorax and pneumomediastinum) in adult patients with COVID-19 interstitial pneumonia. Only patients treated with NIRS outside the ICU were considered. Baseline characteristics, clinical and radiological disease severity, type of ventilatory support used, blood tests and mortality were recorded. Results: In all, 179 patients were included, 60 of them with barotrauma. They were older and had lower BMI than controls (*p* < 0.001 and *p* = 0.045, respectively). Cases had higher respiratory rates and lower PaO_2_/FiO_2_ (*p* = 0.009 and *p* < 0.001). The frequency of barotrauma was 0.3% [0.1–1.3%], with older age being a risk factor for barotrauma (OR 1.06, *p* = 0.015). Alveolar-arterial gradient (A-a) DO_2_ was protective against barotrauma (OR 0.92 [0.87–0.99], *p* = 0.026). Barotrauma required active treatment, with drainage, in only a minority of cases. The type of NIRS was not explicitly related to the development of barotrauma. Still, an escalation of respiratory support from conventional oxygen therapy, high flow nasal cannula to noninvasive respiratory mask was predictive for in-hospital death (OR 15.51, *p* = 0.001). Conclusions: COVIMIX-2 showed a low frequency for barotrauma, around 0.3%. The type of NIRS used seems not to increase this risk. Patients with barotrauma were older, with more severe systemic disease, and showed increased mortality.

## 1. Introduction

The recent severe acute respiratory syndrome coronavirus 2 (SARS-CoV-2) pandemic caused a surge of cases of moderate-to-severe acute respiratory distress syndrome (ARDS) that overwhelmed the intensive care unit (ICU) capacity of local health-care facilities in several regions worldwide and forced clinicians to frequently provide respiratory support outside the ICU setting [1].

The increased respiratory drive seen in severe COVID-19 patients could induce vigorous breathing with uncontrolled inspiratory effort [2,3].

It has been hypothesized, but never proven, that development of barotrauma (spontaneous pneumothorax, pneumomediastinum, or both) may be a marker of P-SILI because spontaneous barotrauma events are considered to be due to excessive transpulmonary pressure gradient [4].

If, on the one hand, it is thought that noninvasive respiratory support could mitigate this detrimental event, on the other hand, improper noninvasive respiratory support could be deleterious [5,6,7].

Recent evidence found that continuous positive airway pressure/pressure support ventilation (C-PAP/PSV), compared with conventional oxygen therapy (COT), increased the risk of barotrauma, while high-flow nasal oxygen (HFNO) did not [8]. These data suggest that NIRS tools may contribute differently to the development of P-SILI with consequent prolonged length of hospital stay and, potentially, worse long-term outcomes [9].

Early identification of patients at risk for barotrauma according to the “Macklin effect” at CT scan, which appears as a linear collection of air tracking along with bronchovascular bundles, visceral pleura and/or interlobular septa, may allow clinicians to implement different NIRS tools and management strategies, such as early invasive ventilation or extracorporeal membrane oxygenation support [10].

Because NIRS before COVID-19 has not been so extensively used outside an ICU setting, we performed this ancillary COVIMIX-2 study to investigate the different respiratory support effects, if any, on barotrauma in patients receiving NIRS outside the ICU who were affected by COVID-19 interstitial pneumonia.

## 2. Materials and Methods

### 2.1. Study Design and Ethics Approval

This was an ancillary analysis of the COVIMIX study [8]. Briefly, the COVIMIX was a multicenter observational study with the main aim of investigating the effect of the different respiratory support strategies on barotrauma occurrence over the entire spectrum of the hospitalization for COVID-19 pneumonia. The original study enrolled patients in 20 Italian hospitals from 1 March 2020 to 28 February 2021. COVIMIX included patients requiring ICU admission as well as patients treated in normal- and intermediate-care wards. Details on the study methodology have been previously published.

### 2.2. Inclusion and Exclusion Criteria

In this ancillary analysis, we considered from the original COVIMIX database all patients who developed barotrauma, defined as spontaneous pneumothorax, spontaneous pneumomediastinum or both, outside the ICU.

Therefore, patients who developed barotrauma requiring ICU admission were not considered for this analysis.

In the COVIMIX study, cases were matched with controls, and matching was performed per period and unit of admission as previously described. In particular, controls were included when considering patients without barotrauma who were admitted in the same week and in the same treatment unit as the ones experiencing barotrauma, respecting all inclusion and exclusion criteria. All patients received standard care according to current clinical practice guidelines and evidence-based recommendations at the time of enrollment.

### 2.3. Data Collection

For all patients, we recorded (1) demographic and anthropometric data; (2) comorbidities; (3) clinical severity of COVID-19 disease (stratified as asymptomatic, mild or moderate illness following World Health Organization [WHO] classification [11], the Quick COVID-19 Severity Index (qCSI) [12] and 4C mortality score [13]); (4) radiological severity of the disease [14]; (5) type of ventilatory support used (COT, HFNO, C-PAP/PSV); (6) parameters of ventilation (PEEP); (7) blood tests; and (8) mortality.

### 2.4. First and Additional Aims

The first study aim is to describe the effect of the different NIRS strategies on barotrauma occurrence outside the ICU. Additional aims describe the overall frequency of barotrauma outside ICU and eventual treatments required. Finally, the characteristics of respiratory failure, blood tests, infections, hospital length of stay and mortality of patients experiencing barotrauma are compared with those of a matched control group to identify possible similarities or important clinical differences.

### 2.5. Statistical Analysis

Categorical variables were presented as absolute values (percentages), and continuous variables were described as either mean and standard deviation or median and ranges, according to the normality of distribution that was assessed using the Shapiro–Wilk test. Categorical variables were compared using the chi-squared test or Fisher’s exact test, while continuous variables were compared using a student t-test or Mann–Whitney U test, as appropriate. Univariable and multivariable conditional logistic regressions were performed to explore which factors were associated with barotrauma and in-hospital death, stratifying by referral centers. A multiple imputation approach was used to account for missing data, replacing missing values with 50 sets of simulated values and adjusting the obtained parameter estimates for missing-data uncertainty. All clinically relevant variables or those that were significant at *p* < 0.05 in univariable analysis were included in the multivariable analysis, taking into account potential collinearities. Overall survival was described according to the Kaplan–Meier approach. Comparisons among survival distributions were performed using the log-rank test. Two-sided p values of less than 0.05 were determined to be statistically significant. Statistical analyses were performed using Stata/IC 17.0 (StataCorp LP, College Station, TX, USA).

## 3. Results

Considering inclusion/exclusion criteria, 180 of the 400 patients in the COVIMIX study were considered for this ancillary analysis. One patient was excluded due to the lack of complete data. Finally, 179 patients were studied, divided into 60 cases and 119 controls.

Table 1 shows the demographic characteristics of the patients and their co-pathologies.

### 3.1. Baseline Characteristics

The characteristics of the patients were similar between cases and controls except for age and BMI. Comorbidities were the same. Severity of COVID-19 disease according to WHO classification was the same (*p* = 0.553), while qCSI and 4C score demonstrated a higher grade of disease severity in the barotrauma group compared with the control group (*p* < 0.001). The majority of cases compared with controls had greater lung involvement on HRCT with Salaffi et al. severity score and higher rate of pulmonary embolism, 6 (5.1%) versus 16 (27.6%) (*p* < 0.001), as shown in Table 2.

Respiratory rate was higher in cases than controls, 22 (18–25) versus 20 (16.5–22), respectively (*p* 0.009).

The PaO2/FiO2 ratio was also worse in cases, 192 (134–281), than controls, 276 (226–310) mmHg, *p* <0.001 (Table 2).

Furthermore, 25 cases (21.7%) and 20 controls (33.9%) received HFNO before barotrauma diagnosis (*p* 0.083); 44 (80%) cases versus 37 controls (32.2%) received CPAP/NIV before barotrauma (*p* <0.001). PEEP level was available in 26 controls and 37 cases, and their value in the barotrauma versus no barotrauma group was similar (10 vs. 8 cmH_2_O, *p* = 0.64) as shown in Table 3.

From a laboratory point of view, cases compared with controls showed higher inflammatory markers such as pro-adrenomedullin, IL-6 and D-dimer than the control group, as shown in Table 4.

Barotrauma was detected 13 (7–20) days after admission. Isolated pneumomediastinum occurred in 33 cases (55%), and isolated pneumothorax in 19 (31.7%). Pneumomediastinum and pneumothorax were present concomitantly in 8 cases (13.3%). Pneumothorax was more frequent on the right side (55.6%) than the left (33.3%); bilateral pneumothorax was recorded in 3 (11.1%) cases. No treatment was required in 38 (63.3%) cases; chest tube drainage was required in 20 (33.3%), and 2 (3.4%) cases needed surgery. The drainage was left in place for a median of 10 (6–18) days.

Patients with barotrauma were hemodynamically stable in the majority of cases (94%). Only 3 patients exhibited hemodynamical instability and needed emergency thoracic drainage. Length of hospital stay (LOS) was 10 (6–16) versus 27 (18–39) days in controls and cases, respectively (*p* < 0.001). Hospital mortality was higher in cases than controls, 24 (40%) vs. 14 (12%), respectively, *p* < 0.001.

### 3.2. Factor Associated with Barotrauma

The frequency of barotrauma excluding critical ill patients was 0.3% [0.1–1.3%] considering only patients from the COVIMIX study who were not admitted to ICU (15,744 patients with COVID-19 pneumonia).

Age was a risk factor for barotrauma OR 1.07 [1.03–1.13] (*p* < 0.001) at the univariable analysis. It also remained significant at the multivariate analysis with OR 1.06 [1.01–1.12, *p* = 0.015]. The Quick COVID-19 Severity Index (qCSI) was shown to be significant only at the univariate OR 1.21 [1.07–1.37, *p* = 0.003]. In noncritically ill patients, the alveolar-arterial gradient (A-a) DO_2_ was significant at the uni- and multivariate analysis with OR 0.95 [0.91–0.99, *p* = 0.029] and OR 0.92 [0.87–0.99, *p* = 0.026], respectively, for barotrauma as shown in Table 5.

The extent (≤50% vs. >50%) of lung involvement at CT scan was significant only at the univariate, in which OR was 3.69 [1.44–9.43, *p* = 0.007].

Compared with COT, HFNO showed an OR of 11.47 95% [2.64–49.80, *p* = 0.001] at the univariate analysis but not at the multivariate. C-PAP/PSV versus COT showed an OR of 9.87 [3.13–31.17, *p* < 0.001]. COT/HFNO versus C-PAP/PSV showed an OR of 5.58 [2.07 vs. 15.09, *p* < 0.001]. Escalation from COT/HFNO/to CPAP/PSV has an OR of 4.00 [1.67–9.62, *p* = 0.002] for the risk of barotrauma. No differences were found at the multivariate analysis. D-dimer was the only one serum marker that remained significant at the multivariate analysis with OR 1.01 [1.00–1.01, *p* = 0.041].

### 3.3. Factor Associated with Death

Age was an independent risk for death with OR 1.14 [1.06–1.22, *p* < 0.001]. Escalation in ventilation was the only variable that still remained significant at multivariate analysis with an OR 15.51 [3.06–78.6] for death (*p* = 0.001) as shown in Table 6.

Overall mortality in the barotrauma group compared with patients without it was 24% versus 14%, respectively (*p* < 0.001).

## 4. Discussion

In this ancillary observational COVI-MIX2 study, firstly, we observed that HFNO and CPAP/PSV compared to COT did not increase the risk of barotrauma in COVID-19 patients treated outside an ICU; secondly, barotrauma frequency was low, near 0.3%; thirdly, the majority of barotrauma events were managed conservatively; fourthly, patients with barotrauma were older, with a more severe systemic disease, and had higher mortality than controls. Finally, as expected, escalation of noninvasive respiratory support increased the risk of death.

The more severe COVID-19 patients required ICU admission and invasive mechanical ventilation [15,16].

Nevertheless, most of the COVID-19 population admitted to the hospital remained outside ICUs and required less invasive respiratory support [17]. Indeed, standard Venturi Mask (COT), high flow nasal cannula (HFNC) or C-PAP/PSV with full face or helmets were largely adopted in medical- or intermediate-care wards [18]. These different tools have different clinical applications based on the severity of the disease, and in case of clinical worsening conditions, they can be used with an escalation approach [19].

However, potential side effects of the different devices should also be considered. In a large observational study that included ICU patients, we recently demonstrated that HFNO compared to COT did not increase the risk of barotrauma, while C-PAP/PSV or IMV did compared to HFNO [8].

Results of the present study outside ICU settings confirm that HFNO seems to be protective regarding the risk of barotrauma. In addition, no significant association with barotrauma was found at multivariate analysis regarding the use of CPAP/PSV. Neither escalation respiratory support provided more risk of barotrauma, although it was strongly associated with an increased risk of in-hospital death (OR 15.51, *p* = 0.001).

Our results are particularly interesting, as VILI has a well-described role in generating barotrauma [20,21,22]. It is possible that CPAP/PSV use limited the respiratory drive in the most severe patients, thereby actually preventing development of P-SILI and subsequent barotrauma. Similarly, it is simply possible that development of barotrauma is a marker of greater disease severity rather than inadequate respiratory support.

We should consider that COVID-19 patients, especially moderate-severe ones, present high respiratory drive carrying the risk of great inspiratory effort [23]. This translates into increased transpulmonary pressure and lung damage potentially culminating in P-SILI [24]. As a result, tailoring the proper respiratory support could become fundamental to reducing as much as possible this risk of lung injury that could manifest with barotrauma. At present, there are only few data and practical tools aiding clinicians in selecting the best respiratory support type for patients with respiratory failure [25,26,27,28]. This is especially true for patients who do not show clear signs of severe respiratory failure requiring immediate institution of invasive ventilation [29,30]. Our study identified older age, higher levels of inflammation and greater lung involvement as potential risk factors for development of barotrauma. Accordingly, extra care should be used when treating patients with these characteristics. In addition, some authors suggested that PaO_2_/FiO_2_ ratio may be inadequate to precisely characterize severity of hypoxemia as suggested by Tobin [31]. Accordingly, we evaluated alveolar-to-arterial oxygen gradient [(Aa) DO_2_] in addition to PaO_2_/FiO_2_ ratio. Although the median value was not different between cases and controls, we found that higher levels of (A-a) DO_2_ were associated with lower risk of barotrauma (OR 0.92, *p* = 0.026). At this point, we should consider that increased (A-a) DO_2_ values could indicate both ventilation–perfusion mismatch or intrapulmonary shunting. Whenever arterial oxygen content does not increase by adding supplementary oxygen to the patient, it is more likely to be a patient that requires earlier invasive ventilation. We argue that patients included in this sub-analysis probably mainly had a problem of ventilation–perfusion mismatch. As a consequence, the simple delivery of supplementary oxygen with noninvasive tools limited an increase in intrapleural pressure swings, benefiting in terms of P-SILI and possible barotrauma events. However, this is only a speculative hypothesis.

Other studies addressed the rate of barotrauma in noninvasively ventilated COVID-19 patients [32]. However, few studies specifically investigated patients receiving NIRS outside the ICU [33,34]. Overall, reported rates of barotrauma for noninvasively ventilated patients range from less than 0.01% to about 14% [35,36]. In our study, we found a lower frequency of barotrauma outside ICUs in COVID-19 patients, of around 0.3%.

Differences between our study and reports from other groups may depend on baseline disease severity as well as on differences in patient screening and selection. For example, Muley et al., who found a rate of pneumomediastinum of 14%, specifically investigated patients with severe-to-critical illness according to World Health Organization disease severity classifications [37]. Studies performed in general COVID-19 populations reported rates in line with our study [38,39].

It is of note that although our results are in agreement with previous findings, we should be aware that we could have missed some barotrauma events in clinically asymptomatic patients. Indeed, as suggested by Dwarakanath et al., one in five patients could have an incidental diagnosis [36].

Notably, the majority of patients with barotrauma did not require any active treatment such as pleural drainage or a similar option in our cohort, as also reported by other authors [34]. This adds evidence to the fact that barotrauma events without worsening clinical condition could advocate for a “wait and observe” attitude.

Our study also confirms that patients with barotrauma had a more severe systemic disease as reflected by higher qCSI, 4C score, lower PaO_2_/FiO_2_ ratio and greater lung involvement at CT scan. These findings raise the question of whether development of barotrauma is related to different types of respiratory support or whether it is rather an expression of the severity of the baseline disease on which additional factors could play a role [40]. This latter issue is particularly important since an escalation approach (COT followed by HFNO and finally by CPAP/PSV) resulted in increasing the mortality risk of these patients, while barotrauma did. Contrasting evidence is available in the literature on this last topic [38,39,41]. A possible interpretation of this finding is that absence of rapid clinical benefit following institution of NIRS should trigger a quickly increased level of respiratory support—for example, by considering early institution of invasive ventilation rather than, for example, testing CPAP/NIV in patients already worsening on HFNO.

Some limitations need to be acknowledged: limited sample size requires careful interpretation and generalizability of the results. In addition, we did not consider the level of respiratory support expertise of physicians that treated the patients. We specifically focused on patients with COVID-19. Accordingly, our findings may not be applicable in other causes of respiratory failure. However, data on NIRS outside ICU for non-COVID-19 ARDS are scarce, and our results may be of help to plan future research on the topic. Finally, we did not consider the level of care provided in every participating hospital (for example, nurse-to-patient ratio). This is an important aspect that could have influenced patients’ outcome.

## 5. Conclusions

The type of noninvasive respiratory support in COVID-19 patients outside ICUs does not seem to increase the risk of barotrauma, a possible life-threatening event that was reported by this study to be near 0.3% in a large population. Barotrauma in more than 50% of cases was treated conservatively without the necessity of a surgical approach. Patients who developed barotrauma were more often older, with a more severe systemic disease, and had higher mortality than those who did not. Whether barotrauma represents an undesired effect of noninvasive ventilation or an expression of a more severe systemic COVID-19 disease still needs to be clarified.

Notwithstanding, if “the truth lies in the middle”, careful clinical evaluation should lead to the determination of instituting the most appropriate respiratory support that, in the case of non-ICU COVID-19 patients, could be a noninvasive approach. Further prospective studies are needed to confirm our finding.

## Figures and Tables

**Table 1:** Patients’ baseline characteristics at hospital admission.

	**Controls** **(*n* = 119)**	**Cases** **(*n* = 60)**	***p*-Value**
**Gender**, *n* (%)			0.389
Male	84 (70.6)	46 (76.7)	
Female	35 (29.4)	14 (23.3)	
**Age**, median (IQR)	67 (54–75)	75 (65.5–80.5)	**<0.001**
**BMI**, median (IQR)	27.7 (24.6–30.8)	26.1 (24.2–27.7)	**0.045**
**Cardiovascular disease**, *n* (%)	46 (38.7)	30 (50)	0.147
**COPD**, *n* (%)	9 (7.6)	7 (11.7)	0.373
**Solid cancer**, *n* (%)	10 (8.4)	4 (6.7)	0.776
**Hematological disease**, *n* (%)	8 (6.7)	4 (6.7)	1.000
**Diabetes**, *n* (%)	26 (21.9)	15 (25)	0.636
**CKD**, *n* (%)	6 (5.1)	7 (11.7)	0.132
**Liver disease**, *n* (%)	6 (5.0)	-	-
**Days from symptoms to hospital admission,** median (IQR)	8 (6–10)	7 (4–10)	0.059

Legend: BMI = Body Mass Index, COPD = Chronic obstructive pulmonary disease, CKD = chronic kidney disease.

**Table 2:** Severity of disease according to different evaluation tools.

	Controls (*n* = 119)	Cases (*n* = 60)	*p*-Value
**WHO**, *n (%)*			0.553
<2	11 (9.2)	3 (5)	
>2	108 (90.7)	51 (85)	
**qCSI**, median (IQR)	2 (0–5)	6 (5–9)	**<0.001**
**4C score**, median (IQR)	9 (6–11)	11 (9–13)	**<0.001**
**PaO_2_/F_i_O_2_ at admission**, median (IQR)	276-2 (226.4–319.0)	192 (134–281)	**<0.001**
**RR**, median (IQR)	20 (16.5–22)	22 (18–25)	**0.009**
**(A-a) DO_2_**, median (IQR)	42.8 (36.8–53.3)	41.5 (28.6–47.8)	0.103
**CT scan involvement**, *n* (%)			**<0.001**
0–24%	20 (16.8)	1 (1.6)	
25–49%	48 (40.3)	7 (11.6)	
50–74%	30 (25.2)	22 (36.6)	
≥75%	14 (12.5)	13 (30.2)	
**Pleural effusion**, *n* (%)	16 (14.2)	4 (6.7)	0.142
**Air Bronchogram**, *n* (%)	36 (33.6)	3 (5)	**<0.001**
**PE**, *n* (%)	6 (5.1)	16 (27.6)	**<0.001**

Legend: WHO = World Health Organization, qCSI = quick COVID-19 Severity Index, P_a_O_2_/F_i_O_2_ = ratio of arterial oxygen partial pressure (P_a_O_2_ in mmHg) to the fraction of inspired oxygen (F_i_O_2_), RR = respiratory rate, (A-a) DO_2_ = alveolar-arterial gradient of oxygen, CT = thorax scan, PE = pulmonary embolism.

**Table 3:** Type and parameters of ventilatory support and characteristics of barotrauma.

	Controls (*n* = 119)	Cases (*n* = 60)	*p*-Value
**Type of ventilatory support**			
Only COT	65 (54.6)	11 (18.3)	**<0.001**
Only HFNO	5 (4.2)	1 (1.7)	
Only CPAP/PSV	17 (14.3)	22 (36.7)	
Escalation COT(HFNO	8 (6.7)	4 (6.7)	
Escalation COT(HFNO(CPAP/PSV	24 (20.2)	22 (36.7)	
**PEEP level during CPAP**	8 [8–10]	8 [7–10]	0.53
**PEEP level during PSV**	10 [8–10]	10 [8–10]	0.51
**PSV level**	8 [6–9]	6 [5–8]	0.28
**Sedation**, *n* (%)	2/33 (6.1)	16/58 (27.6)	**0.013**
**Prone position**, *n* (%)	1/34 (2.9)	22/58 (37.9)	**<0.001**
**Type of barotrauma** *n* (%)			
PMD and PNX		33 (55)	
Pneumomediastinum		19 (31.7)	
Pneumothorax		8 (13.3)	
**Side of barotrauma**, *n* (%)			
Right		15 (55.6)	
Left		9 (33.3)	
Bilateral		3 (11.1)	
**Hemodynamic stability**, *n* (%)			
Stable		56 (94.9)	
Unstable		3 (5.1)	
**PNX extension**, *n* (%)			
Large (≥2 cm)		20 (64.5)	
Small (<2 cm)		11 (34.5)	
**Treatment of barotrauma**, *n* (%)			
Observation only		38 (63.3)	
Drainage		20 (33.3)	
Surgery		2 (3.4)	

Legend: CPAP/PSV = Continuous positive airway pressure ventilation/pressure support ventilation, HFNO = high flow nasal oxygen, COT = conventional oxygen therapy, PMD = pneumomediastinum, PNX = pneumothorax.

**Table 4:** Blood tests and drugs used.

	Controls (*n* = 119)	Cases (*n* = 60)	*p*-Value
**WBC/uL**, median (IQR)	7280 (5120–9580)	7060 (3985–9805)	0.456
**Lymphocyte/μL**, median (IQR)	820 (560–1150)	610 (425–830)	**0.005**
**CRP (mg/L)**, median (IQR)	69.8 (27.7–112.7)	56.3 (22.0–150.5)	0.765
**Procalcitonin**, median (IQR)	0.08 (0.04–0.18)	0.09 (0.06–0.36)	0.051
**Pro-adrenomedullin (mmol/L)**, median (IQR)	0.88 (0.70–1.21)	1.15 (0.80–2.16)	**0.024**
**IL-6 (pg/mL)**, median (IQR)	23.5 (16–40)	50.3 (25.1–118.5)	**0.006**
**LDH (IU/L)**, median (IQR)	502 (382–673)	470 (329–651)	0.481
**D-dimer (FeU/mL)**, median (IQR)	627.5 (446–907)	1083.5 (632.5–4067.5)	**<0.001**
**Antibiotics**, *n* (%)	64 (53.8)	54 (90)	**<0.001**
**Bacterial infection**, *n* (%)	6 (5.0)	4 (6.7)	0.734
**Fungal infection**, *n* (%)	1 (0.8)	5 (8.3)	**0.017**
**Steroids**, *n* (%)	96 (80.7)	58 (96.7)	**0.004**
**Anticoagulants**, *n* (%)	115 (96.6)	56 (93.3)	0.444

Legend: WBC = white blood cell, CRP = C reactive protein, IL-6 = interleukin 6, LDH = lactate dehydrogenase.

**Table 5:** Factors associated with barotrauma (univariable and multivariable analysis).

	Univariable Analysis			Multivariable Analysis		
	OR	95% CI	*p*-Value	OR	95% CI	*p*-Value
**Gender (Male)**	1.60	0.65–3.94	0.303			
**Age**	1.07	1.03–1.13	**<0.001**	1.06	1.01–1.12	**0.015**
**BMI**	0.91	0.83–1.01	0.057			
**Cardiovascular disease**	1.81	0.82–3.97	0.141			
**COPD**	1.37	0.43–4.41	0.593			
**Solid cancer**	0.93	0.24–3.51	0.911			
**Hematologic disease**	0.98	0.25–3.90	0.980			
**Diabetes**	0.95	0.40–2.28	0.908			
**CKD**	2.47	0.68–8.90	0.168			
**Immuno-suppression**	3.55	0.86–14.69	0.081			
**qCSI**	1.21	1.07–1.37	**0.003**			
**P_a_O_2_/F_i_O_2_ at admission**	1.00	0.99–1.01	0.727			
**RR at admission**	1.03	0.96–1.10	0.396			
**(A-a) DO_2_**	0.95	0.91–0.99	**0.029**	0.92	0.87–0.99	**0.026**
**Extent (%) of lung involvement**						
≥50% vs. <50%	3.69	1.44–9.43	0.007	2.49	0.59–10.61	0.216
**Ventilation strategies**						
HFNO vs. COT	11.47	2.64–49.80	**0.001**			
CPAP/PSV vs. COT	9.87	3.13–31.17	**<0.001**			
CPAP/PSV vs. HFNO	0.86	0.21–3.45	0.832			
**CPAP/PSV vs. COT/HNFO**	5.58	2.07–15.09	**0.001**			
**Escalation of ventilatory support vs. non escalation**	4.00	1.67–9.62	**0.002**	2.46	0.69–8.74	0.163
**WBC (*n*/µL)**	1.00	0.99–1.01	0.610			
**Lymphocytes count(*n*/µL)**	1.00	0.99–1.01	0.706			
**CRP (mg/L)**	1.01	0.99–1.01	0.068			
**Procalcitonin (ng/mL)**	2.37	0.89–6.35	0.086			
**Proadrenomedullin (mmol/L)**	1.89	0.59–6.12	0.283			
**IL-6 (pg/mL)**	1.00	0.99–1.01	0.478			
**LDH (IU/L)**	1.01	1.01–1.01	**0.010**	1.00	0.99–1.01	0.058
**D-dimer test (FeU/mL)**	1.01	1.01–1.01	**0.011**	1.01	1.00–1.01	**0.041**
**Bacterial co-infections**	0.34	0.05–2.26	0.266			
**Fungal co-infections**	9.55	0.89–102.97	0.063			

Legend: BMI = Body Mass Index, COPD = Chronic obstructive pulmonary disease, CKD = Chronic kidney disease, qCSI = quick COVID-19 Severity Index, P_a_O_2_/F_i_O_2_ = ratio of arterial oxygen partial pressure (P_a_O_2_ in mmHg) to the fraction of inspired oxygen (F_i_O_2_), RR = respiratory rate, (A-a) DO_2_ = alveolar-arterial gradient of oxygen, CPAP/PSV = Continuous positive airway pressure ventilation/pressure support ventilation, HFNO = high flow nasal oxygen, COT = conventional oxygen therapy, WBC = white blood cell, CRP = C reactive protein, IL-6 = interleukin 6, LDH = lactate dehydrogenase.

**Table 6:** Independent risk factors for in-hospital death.

	Univariable Analysis			Multivariable Analysis		
	OR	95% CI	*p*-Value	OR	95% CI	*p*-Value
**Gender (Male)**	0.33	0.12–0.90	**0.030**	0.37	0.10–1.33	0.128
**Age**	1.13	1.06–1.20	**<0.001**	1.14	1.06–1.22	**<0.001**
**PaO_2_/FiO_2_ ratio at admission**	1.00	0.99–1.01	0.482			
**Respiratory rate at admission**	1.05	0.97–1.13	0.219			
**Barotrauma**	2.76	1.10–6.96	**0.031**	1.45	0.43–4.93	0.554
**CPAP/PSV vs. HFNO/COT**	51.34	6.09–432.71	**<0.001**			
**Escalation in ventilation vs. no escalation**	7.86	2.39–25.88	**0.001**	15.51	3.06–78.67	**0.001**
**Bacterial co-infections**	1.92	0.38–9.80	0.433			
**Fungal co-infections**	1.78	0.26–12.28	0.558			

Legend: P_a_O_2_/F_i_O_2_ = ratio of arterial oxygen partial pressure (P_a_O_2_ in mmHg) to the fraction of inspired oxygen (F_i_O_2_), CPAP/PSV = Continuous positive airway pressure ventilation/pressure support ventilation, HFNO = high flow nasal oxygen, COT = conventional oxygen therapy.

## Data Availability

Data are available upon reasonable request from the corresponding author.
